# A retrospective analysis of bipolar depression treated with transcranial magnetic stimulation

**DOI:** 10.1002/brb3.1805

**Published:** 2020-11-10

**Authors:** Eric L. Goldwaser, Kathy Daddario, Scott T. Aaronson

**Affiliations:** ^1^ Department of Psychiatry University of Maryland Medical Center and Sheppard Pratt Health System Baltimore Maryland USA; ^2^ Clinical Research Sheppard Pratt Health System Baltimore Maryland USA

**Keywords:** bipolar depression, bipolar disorder, neuromodulation, transcranial magnetic stimulation

## Abstract

**Introduction:**

Treatment options are limited for patients with bipolar depression. Antidepressants added to mood stabilizers even carry risks of precipitating mixed/manic episodes. Transcranial magnetic stimulation (TMS) may provide a safe and effective option for these patients.

**Methods:**

Database analysis of the TMS Service at Sheppard Pratt Health System identified patients with bipolar disorder type I (BD1) or II (BD2) in a pure depressive phase at initiation of TMS. Records were reviewed for response and remission rates based on MADRS scores, time to effect, and adverse events, notably treatment‐emergent affective switching. All had failed at least two prior treatments for depression, were currently on at least one mood stabilizer and off antidepressants. Stimulation parameters targeted left dorsolateral prefrontal cortex: 120% motor threshold, 10 pulses per second (pps) × 4s, intertrain interval (ITI) 26s, 75 trains (37.5 min/session) for 3,000 pps total, 5 sessions/week for 30 total treatments, or until remission criteria were met.

**Results:**

A total of 44 patients with BD were identified, representing 15% of the total TMS population. 77% of those who completed a course of TMS met response criteria, and 41% of subjects who completed at least 25 treatments met remission criteria. Subjects with BD1 were more likely to respond, remit, or suffer an adverse event than those with BD2. No patient met clinical criteria for a manic/mixed episode, but four (10%) discontinued due to concerns of activation.

**Conclusions:**

TMS is effective in the bipolar depressed population where episode focused intervention can be specifically offered. Risk of psychomotor agitation must be closely monitored.

## Graphical abstract

A retrospective analysis of 39 patients with bipolar depression treated with a standardized protocol of transcranial magnetic stimulation (TMS) demonstrated a response rate of 77% and a remission rate of 41%. These preliminary data support further exploration of TMS as a possible intervention in this condition for which few treatment options exist.

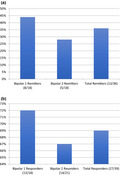

## 1 INTRODUCTION/BACKGROUND

Bipolar disorder represents a heterogeneous group of devastating psychiatric symptoms that can span a gamut of presentations. Differentiating between bipolar disorder type I (BD1) and II (BD2) is no longer considered to denote illness severity per se, as afflicted individuals in both groups share similar degrees of symptom burden, particularly imparted by the chronicity and impairment of the depressive episodes experienced (Judd, Akiskal, et al., 2003; Judd et al., 2002, 2005; Judd, Schettler, et al., 2003). In fact, suicide attempts in both types of bipolar disorder occur without significant differences in rate, and BD2 may actually seek more lethal means (Novick, Swartz, & Frank, 2010).

Despite the recognized high morbidity and mortality, there are a paucity of options for patients afflicted with bipolar depression. The Systemic Treatment Enhancement Program for Bipolar Disorder (STEP‐BD) was designed in 2003 to be able to address questions related to treatment efficacy and operationalize approaches to management of this complex, pervasive, serious mental illness (Sachs et al., 2003). The current FDA‐approved evidence base for the treatment of bipolar depression is limited to four atypical antipsychotics, one of which is paired with an SSRI (Earley et al., 2019; Nierenberg, McIntyre, & Sachs, 2015). The data analyzed from the STEP‐BD study support the notion that the use of antidepressants added to mood stabilizers provides no improved outcome but may, on the contrary, carry the risk of precipitating or supporting a mixed or manic episode, bringing to light not only a question of benefit, but even harm and safety in their provision (El‐Mallakh et al., 2015; Goldberg et al., 2007; Sachs et al., 2007). A problem in developing safe and effective treatment paradigms for bipolar depression is that the population is so heterogeneous and subgroups may require different pharmacologic interventions (Altshuler, Frye, & Gitlin, 2003; Goldberg et al., 2015).

Transcranial magnetic stimulation (TMS) has had a growing impact on the treatment of major depressive disorder (MDD) and unipolar depression. While there is a clear evidence base for the use of TMS in MDD, there is, yet, only a small anecdotal base for its use in bipolar depression (Connolly, Helmer, Cristancho, Cristancho, & O'Reardon, 2012; Noda, Daskalakis, Ramos, & Blumberger, 2013; Wozniak‐Kwasniewska, Szekely, Harquel, Bougerol, & David, 2015), which is further complicated by a wide variety stimulation strategies which has hampered the development of a clear paradigm (McGirr et al., 2016).

A unique aspect of TMS for the bipolar depression patient is the ability to focus the neurostimulation during the depressive episode without providing it as a chronic, daily intervention as is the case with most currently formulated medications. Moreover, the mode of delivery, that is, the necessitating of seeing a provider regularly, also allows for close monitoring in the acute provision of the treatment, affording a unique insight that may promote efficacy and safety if shown to help.

The goal of this retrospective analysis is to provide a foundation for future prospective, open‐label clinical trials looking at rates of efficacy and risk of treatment‐emergent mania or affective switching. Our primary hypothesis was that TMS would improve symptom‐burden evinced by the MADRS for BD patients while in the throes of a depressive episode and that this would be present across both BD1 and BD2 subtypes. Our secondary hypothesis was that TMS would provide a safe avenue for treatment management without an added risk of manic conversion.

## 2 METHODS

### 2.1 Participants

This study is a retrospective review of the clinical records of patients who were evaluated and treated by the Transcranial Magnetic Stimulation (TMS) service at a large, primary psychiatric, teaching hospital, Sheppard Pratt Health System, in Baltimore, Maryland, between April 2010 and December 2015. As part of routine clinical care, all patients referred to TMS are seen and evaluated by a senior clinician where a diagnosis is made or confirmed and patients’ candidacy and appropriateness are determined for provision of TMS. Patients with a diagnosis of bipolar disorder are required to be on at least one mood stabilizer at an effective dose for at least two weeks and were taken off antidepressants prior to TMS treatment. All patients have Montgomery‐Asberg Depression Rating Scale (MADRS) interviews at the start and finish of the course of TMS treatments as a standard of practice. Treatments were given by experienced psychiatric nurses who evaluated patients at all visits.

Upon review of records available, 44 patients (about 15% of the entire population of patients receiving TMS) were diagnosed with bipolar disorder and in the depressed phase. Details about diagnosis (i.e., BD1 or BD2), history of treatment resistance, and reviews of depression severity by MADRS score were collected and analyzed. Particular attention was paid to rates of response and remission. Response was defined operationally as a 50% improvement in MADRS score from baseline, and remission as an overall MADRS score of 10 or less. Partial response was a final MADRS score with a 25%–49% drop from baseline. For response and remission rates, dropouts due to treatment‐related adverse events are included. Five patients with an inadequate course of treatment due to finishing treatment elsewhere were excluded in the analysis presented, accounting for the 39 patients ultimately included.

### 2.2 TMS protocol

All patients were treated with a solid core figure 8 coil manufactured by Neuronetics, Inc. Stimulation target was over the left dorsolateral prefrontal cortex at 120% magnetic field intensity relative to the subject's resting motor threshold (RMT), at 10 pulses per second (pps) for 4 s, with an off time, or intertrain interval (ITI), of 26 s. Coil was positioned at the F3 location as determined by the standard 10–20 system for electroencephalography (Klem, Luders, Jasper, & Elger, 1999). Prophylactic use of acetaminophen or ibuprofen was allowed for subjects reporting sensations at or near the stimulation site which were uncomfortable or painful. During the first week of treatment only, in the event that the subject was unable to tolerate the treatment at these dose parameters, dose intensity was titrated down to 110% of the RMT, with all other dose parameters remaining the same. Treatment sessions lasted for 37.5 min (75 trains) to provide a total of 3,000 pps. The TMS course was five times a week for a total of 30 treatments or until remission criteria was met.

### 2.3 Rating

The primary objective efficacy measure of this study was a change from baseline to endpoint in MADRS score. The MADRS is a widely used rating scale for severity of mood‐related symptoms (Montgomery and Asberg, 1979). The scale consists of 10 items and ranges from 0 to 60, where higher scores denote more severe symptoms. The MADRS score (as opposed to the alternatively and commonly used Hamilton Rating Scale for Depression) is sensitive to detect changes in the psychic rather than physical symptoms of depression (Carmody et al., 2006). A secondary outcome was the percentage of patients who meet criteria for onset of manic symptoms based on the Young Mania Rating Scale (YMRS; Young, Biggs, Ziegler, & Meyer, 1978). A score of greater than 12 was considered significant for new‐onset mania. The patients were separated based on their diagnostic category of BD1 or BD2, as well as inclusively, in the graphical representations used herein.

### 2.4 Ethical statement

As this article represents a review of outcomes for routine clinical care and not original research, no ethical review was required by the authors’ institution.

## 3 RESULTS

**Figure 1:**
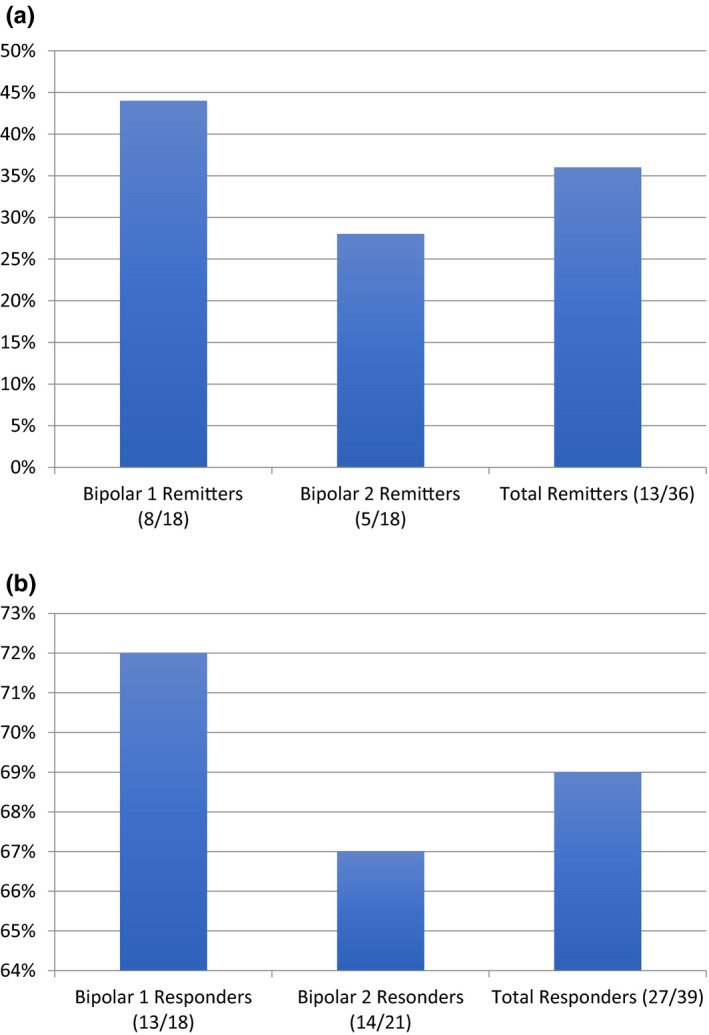
(a) Percent of patients meeting remission by MADRS (MADRS ≤ 10). (b) Percent of patients meeting response by MADRS (50% drop from baseline)

Forty‐four patients were identified in total to be experiencing bipolar depression and receiving TMS. Of these, five were excluded based on not completing the course of treatment on site. The remaining 39 patients with bipolar depression had either an adequate course of treatment or were discontinued due to physician decision about possible activation and side effects, but, importantly, none (0/39) had met criteria for a mixed or manic state (YMRS > 12). Effect of TMS treatment on MADRS scores is graphed in Figure 1 for both remitters (Figure 1a) and responders (Figure 1b), depicted in diagnostic categories of bipolar 1 or bipolar 2, and grouped together.

An adverse event of overstimulation concerning for hypomanic activation was seen in 3/18 BD1 patients (17%) and 1/21 (5%) of BD2 patients for an overall 10% incidence. This adverse event happened early in the course of treatment. For BD1, concern for affective switching occurred at session 5 for two patients and session 9 for one patient, and for BD2 at session 11 for one patient.

In terms of time lapsed from treatment onset to remission in remitters, the average number of sessions needed was 22.5 for BD1 and 22.6 for BD2. Interestingly, six patients had a history of ECT treatment: five patients with BD2 and one patient with BD1. The patient with BD1 had a partial response to a full course of TMS. Of the BD2 patients, two did not respond at all, one was stopped due to an adverse event, one had remitted completely, and one had a partial response.

Four of the total 44 patients identified had their course stopped by their clinician due to side effects. As mentioned, however, importantly none of them met the criteria for mania or mixed state. Five patients had their course completed by a different TMS provider (closer to their home). For the 35 patients who completed a course of treatment, 27 (77%) met response criteria. 32 patients completed at least 25 treatments, of which 13 met remission criteria (41%).

Differentiating BD1 from BD2 in terms of effect between remitters and responders, BD1 showed overall better efficacy. Patients with BD1 had 44% rate of remission and 72% rate of response, while BD2 patients had 28% and 67% rates, respectively.

## 4 DISCUSSION

TMS is an increasingly accepted neurostimulation‐based treatment for MDD. While there is growing anecdotal database supporting its use in bipolar depression, high‐quality open‐label efficacy data and safety profiles are still needed for more widespread use and acceptance. Toward this goal, we studied available clinical charts at the TMS service of a large, academic, primary psychiatric hospital for patients with diagnoses of either bipolar disorder type I (BD1) or II (BD2).

For bipolar disorder overall, 77% met response criteria and 41% met remission criteria. These results are numerically superior to our experience in unipolar depression where our response rate is 62% and our remission rate is 31%. In general, the treatment has been well tolerated and effective. No treatment‐emergent affective switch was recorded based on clinical criteria, despite concerns that prompted a discontinuation in four of the subjects with a subclinical degree of activation, roughly 10% of all bipolar patients. Most of these patients have a long history of either poor efficacy or safety issues with the use of antidepressants and inadequate antidepressant response with the use of mood stabilizers or atypical antipsychotics alone.

With these results, we are able to add to the literature supporting use of TMS in bipolar depression. Refinement and consensus agreement are needed with regard to the method of delivery, however. Given that the FDA has already approved the left dorsolateral prefrontal cortex (DLPFC) for use in unipolar treatment‐resistant depression, this can be readily advantageous in similarly leveraging for use in treatment‐resistant bipolar depression. However, studies have explored use of 1 Hz frequency delivery to the right DLPFC to achieve similar results of depression remission (Dell'Osso et al., 2009, 2015). Parameters surrounding methods in which TMS is administered can be directed for achieving either neuronal circuitry excitation when provided at “high frequencies” (for instance, 5 Hz and greater) or neuronal inhibition or suppressive changes at “low frequencies” (for instance, 1 Hz and lower; Hallett, 2007; Wagner, Valero‐Cabre, & Pascual‐Leone, 2007). Underlying neurobiological investigations had revealed distinctive functional patterns across hemispheric cortical areas that helped build the model for which TMS settings were then delivered (Kazemi et al., 2016; Nahas, Kozel, Li, Anderson, & George, 2003; Nahas et al., 1999).

Absolute consistency across TMS provision methods seems difficult to achieve given the paucity of such in the current literature. For this reason, it is all the more important that large‐scale studies be performed to establish optimal, and even acceptable, parameters. Although studies exist that report less significant, or even null findings with TMS and bipolar depression (Fitzgerald et al., 2016; Nahas et al., 2003), the comparability from a methodological concern becomes paramount again. For instance, in these instances of “negative results,” the active pulses delivered per session appeared markedly lower than typically used, and total duration of treatment course and number of sessions may be as low as two weeks total. Interestingly, another report found that no treatment separation was found actually during four weeks of administration, and treatment effects only began to present by six weeks, that is after completion of the TMS course (Tamas, Menkes, & El‐Mallakh, 2007), again pointing to lack of consistency in the way in which these studies are able to be compared, and may seriously impact the results and interpretation as inadequate or in error.

One study with similar treatment parameters as ours (i.e., left DLPFC, 120% MT, 10 Hz, at least 2000 pps) found 60% response rate, using the MADRS, of TMS in their BD patients (Wozniak‐Kwasniewska et al., 2015). Still though, intertrain intervals, pulses per session delivered in total, and number of sessions all slightly differed, naturally limiting generalizability even in the most comparable of studies performed, asking and attempting to answer the same question at stake. Another group performed a retrospective review of twenty patients treated with TMS to the left DLPFC, (120% MT, 10 Hz) and found only a 35% response rate and 15% remission rate, however, had used clinical global impression (CGI) scores for their population psychometrics reported (Connolly et al., 2012). In this cohort, medications were not controlled other than not being changed. Many other parameters are of note when comparing studies, including type of coil used in the TMS device, method in which the target site is discovered, and even the degree of treatment resistance and responsiveness to ECT. In light of some of the more recent evidences gathered from accruing reports, case series, and clinical trials, certainly two weeks (and even up to four weeks) of a timeframe to build efficacy may be a costly underestimate, and likewise the pulses delivered per session if under 2000, frequency of delivery, and others, will need to be further characterized and detailed in order to direct future studies to truly answer the question of TMS’ use in BD.

One prospective study found a 63% response and 52% remission rate in their 19 patient sample sizes, by targeting the left DLPFC at 120% MT with 20 Hz frequency using the H‐coil design (Harel et al., 2011). In agreement with these findings, a meta‐analysis of 19 randomized clinical trials using TMS in 181 total bipolar depression patients found that overall treatment efficacy was observed to be comparable across MDD and BD, and was safe with regard to treatment‐emergent affective switches (McGirr et al., 2016). However, the authors noted that the designs and methods varied greatly across the studies, limiting generalizability even from one randomized clinical trial to the next. Despite certain important differences, clinical response was achieved by roughly 44% of patients receiving active TMS compared to 25% receiving sham treatment. Similar optimism was shared in another study using the HAMD in a retrospective design, in which 34% of patients with BD met response criteria and 26% met remission criteria, and no manic switches were observed during the treatment or in the weeks following treatment conclusion (Carnell, Clarke, Gill, & Galletly, 2017). Moreover, no separations in efficacy were observed between BD1 or BD2.

The treatment goals and symptom relief that TMS may offer for this difficult to manage population are becoming more within reach with the concerted efforts of studies from researchers like this one. Some important ways that TMS appears to offer new hope to BD patients include a shorter time to response in the pervasive depressive phases that plague treatment‐resistant patients, especially compared with the available data in unipolar depressed patients. Moreover, an important advantage is that it can be safely given in bipolar depression without inducing a manic or mixed episode switch in patients who are receiving adequate concurrent mood stabilization medication. Our results here support further investigations into the efficacy of TMS in BD, which, if verified and generalized, may redefine treatment algorithms in a subset of responsive patients to neuromodulation. However, the development of thoughtful, consistent parameters for treatment is essential and likely needs to include clear diagnosis (bipolar disorder in the depressive phase), a stable platform of medications (we suggest at least one mood stabilizer and no antidepressants) and a unified approach to the TMS stimulation (we suggest activation stimulation at the left DLPFC).

Fascinatingly, and somewhat surprisingly, bipolar depression may be a better target than unipolar depression given the strikingly high response rate observed in our cohort; however, patient selection and proper clinical documentation and assessment are crucial. Ensuring adequate mood stabilization is critical to display stability and perhaps may even prove to lower risks associated with affective switching and response rate benefits—moreover, there may be differences in these measurements for BD2 compared to BD1.

Limitations of this study exist inherent to methodologic concerns in retrospective chart data gathering. For one, we are unable to more fully discern qualitative aspects of TMS effects outside of the few parameters captured by the MADRS. Quality of life measures have been shown to be improved in neuromodulation‐responsive populations of affective disorder patients, even when null improvements are reported with standardized depression rating scales (Conway et al., 2018), suggesting there may be an underestimation of the actual benefits TMS may have. Moreover, confounds such as differential psychopharmacology and medical comorbidities were not accounted for as covariates in biostatistical considerations, as was not the aim of this report, but would certainly be needed for future, larger‐scale validation studies addressing this question.

## 5 CONCLUSION

Results reported here suggest that TMS for bipolar depression is both more successful and more prone to adverse events than a similar unipolar population. To this end, a higher percentage of bipolar patients responded to TMS and required fewer treatments to see an effect than for unipolar patients in our experience. This higher risk–benefit ratio is greater for BD1 than BD2 patients.

While these results support the continued development of TMS for bipolar depression, care must be taken to make sure patients are adequately mood stabilized and fully in the depressive phase of the illness before commencing treatment, despite the low probability of affective switching.

Much is left to be deciphered in this exciting area of neuromodulation, and a larger, open‐label prospective study would be more able to address some of the yet unanswered and vital questions remaining prior to pursuing mainstay administration of TMS to bipolar depressed patients.

## CONFLICT OF INTEREST

STA is a consultant to Neuronetics, LivaNova, Janssen, Genomind, and Sage Therapeutics and has received honoraria from Sunovion and Janssen. He also has research support from Neuronetics and Compass Pathways. KD and ELG have no financial interests to disclose.

## AUTHOR CONTRIBUTIONS

Dr. Goldwaser wrote several drafts of the manuscript. Ms. Daddario performed most of the TMS treatment session and did primary data collection and analysis. Dr. Aaronson was the treating clinician for all the patients, wrote the original manuscript, edited the final manuscript, and did the statistical analyses.

### Peer Review

The peer review history for this article is available at https://publons.com/publon/10.1002/brb3.1805.

## Data Availability

The data are all available from the corresponding author upon request.
